# Comparison of clinical features and prognosis in patients with cryptogenic and secondary organizing pneumonia

**DOI:** 10.1186/s12890-021-01707-z

**Published:** 2021-10-29

**Authors:** Keum-Ju Choi, Eun-Hyung Yoo, Kyung Chan Kim, Eun Jin Kim

**Affiliations:** 1 Department of Internal Medicine, Daegu Catholic University Medical Center, Daegu Catholic University School of Medicine, 33, Duryugongwon-ro 17-gil, Nam-gu, Daegu, 42472 South Korea; 2 Department of Laboratory Medicine, Daegu Catholic University Medical Center, Daegu Catholic University School of Medicine, Daegu, South Korea

**Keywords:** Organizing pneumonia, Cryptogenic organizing pneumonia, Secondary organizing pneumonia, Infection, Bronchoalveolar lavage

## Abstract

**Background:**

Organizing pneumonia (OP) can be diagnosed pathologically, and cryptogenic OP (COP) and secondary OP (SOP) have been classified by cause and particular underlying context. Because it is clinically difficult to differentiate between COP and SOP, this study investigated characteristics that could distinguish between COP and SOP.

**Methods:**

The medical records of patients who underwent lung biopsy for a diagnosis of OP at a single tertiary hospital from January 2016 to December 2018 were retrospectively reviewed.

**Results:**

Eighty-five patients had pathologically proven OP, including 16 diagnosed with COP and 69 diagnosed with SOP. The most common cause of SOP was infectious pneumonia, observed in 57 (82.6%) of the 69 patients, followed by cancer and radiation pneumonitis. The pathogens causing infectious pneumonia were identified in 45 (65.2%) patients. There were no differences in age, sex, and lung function between the COP and SOP groups. Median body mass index was significantly lower (*P* = 0.030), and median time from symptom onset to hospital admission significantly shorter (*P* = 0.006), in the SOP than in the COP group. Fever was more common in the SOP group (*P* = 0.024), and CURB 65, an index of pneumonia severity, tended to be higher in the SOP group (*P* = 0.017). Some laboratory results differed significantly between the two groups. Lymphocyte counts in bronchoalveolar lavage (BAL) fluid were significantly higher in the COP than in the SOP group (*P* = 0.012). Radiologic findings showed that effusion was more common in the SOP group (*P* = 0.036). There were no between-group differences in steroid use, 30 day and in-hospital mortality rates, and rates of OP outcomes and recurrences. Pneumonia recurrence rate was significantly higher in SOP patients who were than were not treated with steroids (*P* = 0.035).

**Conclusions:**

Infection is the main cause of SOP. Symptom onset is more rapid in patients with SOP than with COP. Some blood and BAL fluid test results differed significantly in the COP and SOP groups. Pleural effusion was more common in the SOP group but there were no differences in clinical course. Recurrence in patients with SOP was more common in those who were than were not treated with steroids.

## Background

Organizing pneumonia (OP) is an interstitial lung disease histologically characterized by patchy filling of the lung alveoli and respiratory bronchioles by loose plugs of granulation tissue [1, 2]. OP was first described histologically during the early 20th century [3] and has been regarded as a distinct pathological entity since the 1980s [1, 4]. Histological, clinical, and radiological advances have increased understanding of OP, which is presently classified as an interstitial lung disease [5, 6].

OP is initiated by lung injuries, with the alveolar epithelium reacting to produce granulation tissue [6]. Despite having the same pathologic patterns, OP can be classified as either cryptogenic OP (COP) or secondary OP (SOP). SOP can be caused by infection, drug toxicity, drug intoxication, inhalation of toxic gases, aspiration of gastric contents, connective tissue diseases (CTD), organ transplantation, radiotherapy, vasculitis, tumors, pulmonary infarction, hypersensitivity pneumonitis, eosinophilic pneumonia, and other interstitial lung diseases [2, 7]. COP can be diagnosed by excluding SOP, based on clinical history, characteristics, laboratory tests, radiologic findings, and associated pathologic findings [8, 9]. Clinical confusion has arisen in the differential diagnosis of COP or SOP, as differences in their characteristics have not been clearly identified [10–15]. The present study investigated whether any characteristics could distinguish between COP and SOP. The causes, clinical characteristics, laboratory results, radiologic findings, and prognosis were therefore compared in patients with pathologically proven SOP and COP.

## Methods

### Study design

**Fig. 1:**
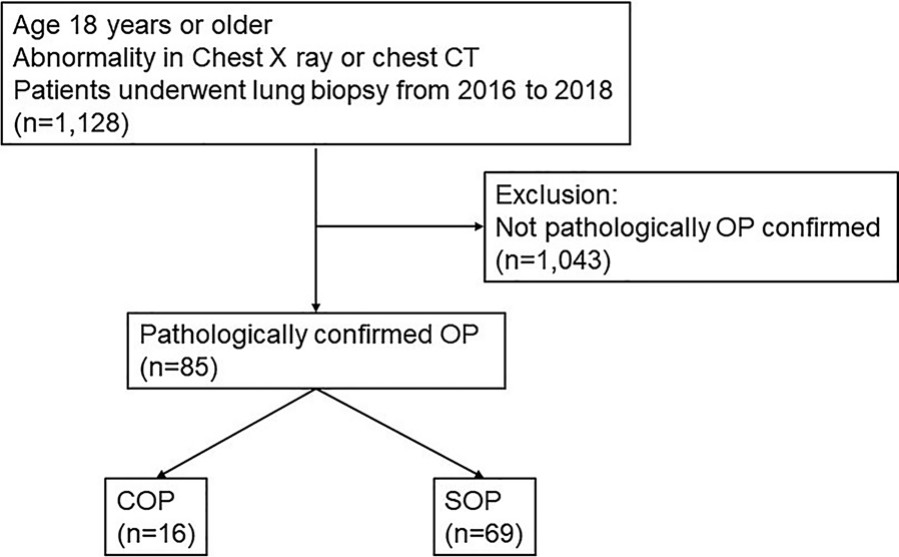
Flow chart of the inclusion and exclusion criteria and study design. *CT* computed tomography, *COP* cryptogenic organizing pneumonia, *SOP* secondary organizing pneumonia, *OP* organizing pneumonia

The medical records of all patients who underwent lung biopsy at a single tertiary medical institution, Daegu Catholic University Medical Center, South Korea, from January 2016 to December 2018 were retrospectively reviewed. OP was diagnosed by the presence of intraluminal granulation tissue in the alveolar ducts, alveoli, and bronchiolar lumen, as confirmed pathologically by lung biopsy results. This study included (1) cases with abnormal features in chest X ray or chest computed tomography (CT) in adults 18 years of age or older, and (2) cases diagnosed with OP through lung biopsy. In this study, cases where biopsy could not be performed, or OP was not confirmed by biopsy were excluded (Fig. 1). A total of 85 patients were identified.

Basic clinical characteristics, including clinical history, symptoms, comorbidities, and concomitant drug use; radiologic findings, including CT results; biopsy methods and patterns, laboratory data, results of sputum and bronchioloalveolar lavage (BAL) fluid tests, culture findings, and polymerase chain reaction (PCR) results on admission and at biopsy were recorded, as were the results of pulmonary function tests (PFT), treatment-associated data, and follow-up data. The clinical diagnosis was confirmed before the biopsy was performed. The predominant pattern of each biopsy specimen was also determined. The patients evaluated the possibility of CTD based on their medical history and symptoms, and whether laboratory test such as muscle enzyme levels were elevated. For only patients with possible CTD or COP, rheumatoid factor, antinuclear antibody, antineutrophil cytoplasmic antibodies, and anti-cyclic citrullinated peptide antibodies were identified. Patients were referred to a rheumatologist when CTD was considered possible.

As this is a retrospective study, the possibility of unchecked laboratory tests or histories may have resulted in selection bias from data missing at random without any intention [16]. To reduce bias, we reconfirmed medical history by telephone in living and reachable patients.

The study protocol was approved by the Institutional Review Board of Daegu Catholic University Hospital (IRB No. CR-21-097).

### Definitions

COP was defined as OP in the absence of an established clinical cause, based on correlations of underlying diseases, concomitant drugs, and microbiological data with a predominant histological pattern. SOP was defined as OP secondary to lung injury of known etiology. The chest CT findings of OP were classified as follows; consolidation, ground glass opacity (GGO), mixed of consolidation and GGO, nodule or mass, perilobular opacity, parenchymal bands or fibrotic pattern, reversed halo sign, mediastinal lymphadenopathy and pleural effusion [6, 17, 18]. Perilobular opacity was defined as curved or polygonal opacities with poorly defined margins around the interlobular septa. Parenchymal bands were defined as extending in a radial manner along the bronchi towards the pleura. Reverse halo sign was defined as a focal rounded area of GGO surrounded by ring or crescent shaped consolidation. Mediastinal lymphadenopathy was defined as mediastinal lymph node size greater than 1 centimeter in short-axis diameter. We also specifically identified the focal OP defined as a solitary focal and nodular opacity [19–21]. Recurrence was defined as worsening of chest X ray after prior improvement; in such cases, re-biopsy was not performed.

### Methods

#### Lung biopsy

Lung tissue samples were obtained by bronchoscopic transbronchial lung biopsy (TBLB), CT-guided lung biopsy, or video-associated surgical lung biopsy. Patients with OP diagnosed by TBLB or CT-guided lung biopsy did not require surgical lung biopsy. Of the 85 patients, 77 (90.6%) were diagnosed by TBLB, including 59 (68.2%) by radial probe and 19 (22.4%) by blind TBLB. Seven (8.2%) patients underwent CT-guided lung biopsy and one (1.2%) underwent surgical lung biopsy. All patients underwent bronchoscopy, even those who underwent CT-guided or surgical lung biopsy, with BAL fluid samples obtained from 43 (50.6%) patients.

#### Microbiological and molecular methods

The quality of sputum and BAL fluid specimens was assessed microscopically using Gram stain smears, as determined by the Murray–Washington grouping system [22]. Specimens were processed and cultured according to standard laboratory procedures. The most purulent portion of each specimen was inoculated onto blood, chocolate, and MacConkey’s agar plates, and incubated at 35 °C in 5% CO_2_ for 48 h. Agar plates were examined at 16–24 and 48 h, and the predominant organisms were identified and quantified. Biochemical identification and antibiotic susceptibility tests were performed using the VITEK 2 System (bioMérieux, Durham, NC, USA) or Microscan WalkAway 96 SI Plus (Beckman Coulter, Brea, CA, USA) according to the manufacturer's instructions. In some cases, sputum, bronchial washing, and BAL fluid samples were subjected to Acid fast bacilli (AFB) smear and culture and fungal culture.

*Mycoplasma pneumoniae, Legionella pneumophila, Streptococcus pneumoniae, Hemophilus influenzae, Bordetella pertussis, and Chlamydophila pneumoniae* were detected in respiratory samples by multiplex real-time PCR tests. To detect viruses, RNA was extracted from clinical samples with Nextractor NX-48 kits (Biosewoom, Seoul, Korea) according to the manufacturer’s instructions. Real-time RT-PCR assays were performed to detect 14 respiratory viruses (adenovirus; human rhinovirus, human coronaviruses (CoV) NL63/HKU1, 229E, and OC43; influenza A and B viruses; parainfluenza viruses (PIV) 1, 2, and 3; respiratory syncytial viruses A and B; human bocavirus, and metapneumovirus) using Real-Q RV Detection kits (Biosewoom) and the CFX96 Real-Time System (Bio-Rad, Hercules, CA, USA). Respiratory samples were also analyzed using the BioFire™ Diagnostics Respiratory Panel and FilmArray™ multiplex PCR System (bioMérieux) to detect the following pathogens: adenovirus, CoV 229E, CoV HKU1, CoV NL63, CoV OC43, human metapneumovirus, human rhinovirus/enterovirus, influenza A H1 virus, influenza A H1 2009 virus, influenza A H3 virus, influenza B virus, parainfluenza virus 1, parainfluenza virus 2, parainfluenza virus 3, parainfluenza virus 4, respiratory syncytial virus, *Bordetella pertussis*, *Chlamydophila pneumoniae*, and *Mycoplasma pneumoniae*.

#### PFT and chest CT

Forty-one (48.2%) patients were assessed by PFTs, with results reported as absolute values and % of predicted values. PFT results were interpreted as normal, restrictive, obstructive, and mixed patterns according to American Thoracic Society criteria [23].

All patients were evaluated by chest CT to assess the patterns and distribution of pulmonary abnormalities prior to lung biopsy. Based on chest CT, pulmonary patterns were classified as consolidation, GGO, mixed of consolidation and GGO, nodule or mass, perilobular opacity, parenchymal bands or fibrotic pattern, reversed halo sign, mediastinal lymphadenopathy, pleural effusion [6, 17, 18], cavity and focal solitary nodule [19–21].

#### Treatment and follow-up

Each patient was started on treatment with antibiotics and steroids at the discretion of each physician based on the clinical condition of the patient. Most patients were started on methylprednisolone 0.75 mg/kg/day or higher [8, 24], with the duration and dose depending on the judgment of each physician. Patients were followed-up and recurrence monitored for at least 2 years. When following up the patients, chest X-ray was checked in all patients, but chest CT was performed at the physician's discretion.

### Statistical analysis

Normally distributed continuous variables were expressed as numbers and percentages or as means or medians and interquartile ranges (IQRs), whereas non-normally distributed continuous variables were expressed as medians and IQRs. Results in the COP and SOP groups were compared by Mann–Whitney U-tests. Qualitative variables between the two groups were compared using chi-square (χ2) tests or Fisher’s exact tests. CURB-65 and the number of affected lobes on chest CT between the two groups were compared using linear-by-linear association. All statistical analyses were performed using SPSS version 25 (SPSS, Inc., Chicago, IL, USA), with statistical significance set at *P *< 0.05.

## Results

Of the 85 patients with pathologically confirmed OP on lung biopsy, 16 (18.8%) were diagnosed with COP and 69 (81.2%) with SOP. Infectious pneumonia was the most frequent cause of SOP, occurring in 57 patients (82.6%), followed by cancer and radiation pneumonitis. The bacteria causing infectious pneumonia were confirmed in 45 patients (65.2%), either by culture or PCR testing. *Acinetobacter baumanii* was the most frequently identified, followed by *Stenotrophomonas*, *Tuberculosis*, *Streptococcus*, *Staphylococcus*, *Klebsiella*, and fungi. In one patient each, SOP was caused by CTD, hypersensitivity pneumonitis, and chronic eosinophilic pneumonia, and was drug (adalimumab)-related in one patient (Table 1).

**Table 1:** Diseases associated with secondary organizing pneumonia

Associated diseases (n = 69)	No. (%)
Infectious pneumonia including lung abscess or aspiration pneumonia	57 (82.6)
Acinetobacter	8 (11.6)
Tuberculosis	7 (10.1)
Stenotrophomonas	7 (10.1)
Streptococcus	6 (9.7)
Staphylococcus	5 (7.2)
Klebsiella	4 (5.8)
Fungus infection	3 (4.3)
Pneumocystis jiroveci pneumonia	1 (1.4)
Pseudomonas	1 (1.4)
Enterobacter	1 (1.4)
Influenza virus	1 (1.4)
Hemophilus influenzae	1 (1.4)
Cancer	6 (8.7)
Radiation pneumonitis	2 (2.9)
Connected tissue disease	1 (1.4)
Hypersensitivity pneumonitis	1 (1.4)
Drug (adalimumab)	1 (1.4)
Chronic eosinophilic pneumonia	1 (1.4)

Median ages of patients in the COP and SOP groups were 64 and 71 years, respectively, with no significant between-group difference. The percentages of men were higher than those of women in both groups, but the difference in gender distribution was not significant. Reasons for biopsy included the pneumonia pattern in 61 patients (71.8%) and the mass form in 24 (28.2%). Median body mass index (BMI) was significantly higher in the COP than in the SOP group (24.1 kg/m^2^ vs. 21.7 kg/m^2^, *P* = 0.030). The median time from the first visit to diagnosis was 13 days in both groups, whereas the median time from symptom onset to hospital admission was significantly longer in the COP than in the SOP group (4 weeks vs. 1 week, *P* = 0.006). Fever was significantly more common in the SOP group (*P* = 0.024). The most common symptoms in all patients were cough, sputum, shortness of breath, fever, hemoptysis, and chest pain. Chronic liver disease was significantly more frequent in the COP group (*P* = 0.020). There was a significant difference in CURB-65 score [25], an index of the severity of pneumonia (*P* = 0.017) (Table 2).

**Table 2:** Baseline demographic and clinical characteristics of patients with cryptogenic and secondary organizing pneumonia

	COP (n = 16)	SOP (n = 69)	Total OP (n = 85)	*P*
Age, years	64 (62–74)	71 (61–78)	70.0 (61–77)	0.303
Male:female	11:5	58:11	69:16	0.158
Smoking				0.533
Non-smoker	6 (37.5)	25 (36.2)	31 (36.5)	
Ex-smoker	6 (37.5)	34 (49.3)	40 (47.1)	
Smoker	4 (25.0)	10 (14.5)	14 (16.5)	
Reason for biopsy				0.126
Pneumonic lesion	9 (56.3)	52 (75.4)	61 (71.8)	
Mass lesion	7 (43.8)	17 (24.6)	24 (28.2)	
Duration between first visit and biopsy, days	13 (9–23)	13 (8–20)	13 (8–20)	0.875
BMI, kg/m^2^	24.1 (21.3–25.5)	21.7 (19.7–24.2)	22.0 (19.8–24.5)	0.030**
Symptom duration, weeks	4 (2–5) (n = 13)	1 (1–4) (n = 63)	2 (1–4) (n = 76)	0.006**
Symptom				
Cough	12 (75.0)	50 (72.5)	62 (72.9)	0.837
Sputum	9 (56.3)	37 (53.6)	46 (54.1)	0.849
Dyspnea	9 (56.3)	25 (36.2)	34 (40.0)	0.141
Fever	2 (6.3)	30 (43.5)	32 (37.6)	0.024*
Hemoptysis	2 (12.5)	6 (8.7)	8 (9.4)	0.641
Chest pain	3 (18.8)	3 (4.3)	6 (7.1)	0.078
Mental status change	0	3 (4.3)	3 (3.5)	0.539
Underlying diseases				
Hypertension	4 (25.0)	31 (44.9)	35 (41.2)	0.170
Diabetes mellitus	4 (25.0)	22 (31.9)	26 (30.6)	0.766
Chronic airway disease	3 (18.8)	15 (21.7)	18 (21.2)	0.547
Ischemic heart disease	2 (12.5)	11 (15.9)	13 (15.3)	0.539
Cerebrovascular disease	0	11 (15.9)	11 (12.9)	0.115
Active cancer	1 (6.3)	7 (10.1)	8 (9.4)	0.532
Immune suppression	0	7 (10.1)	7 (8.2)	0.338
Bed bound state	0	7 (10.1)	7 (8.2)	0.338
Atrial fibrillation	2 (12.5)	4 (5.8)	6 (7.1)	0.315
Chronic kidney disease	0	5 (7.2)	5 (5.9)	0.578
Chronic liver disease	3 (18.8)	1 (1.4)	4 (4.7)	0.020*
Other interstitial lung diseases	0	1 (1.4)	1 (1.2)	0.812
Connective tissue diseases	0	1 (1.4)	1 (1.2)	0.812
CURB-65 [25]				0.017***
0	7 (43.8)	12 (17.4)	19(22.4)	
1	7 (43.8)	29 (42.0)	36(42.4)	
2	2 (12.5)	20 (29.0)	22(25.9)	
3	0	7 (10.1)	7(8.2)	
4	0	1 (1.4)	1(1.2)	

Data are presented as number (%) or median (interquartile range [IQR])

*COP* cryptogenic organizing pneumonia, *SOP *secondary organizing pneumonia, *OP* organizing pneumonia, *BMI *body mass index

*P < 0.05 by chi-square tests or Fisher’s exact tests for comparisons between the COP and SOP groups

**P < 0.05 by Mann–Whitney U-tests for comparisons between the COP and SOP groups

***P < 0.05 by linear-by-linear association for comparisons between the COP and SOP groups

Vital signs and PF ratios did not differ between the two groups, nor did leukocyte and neutrophil counts and concentrations of C-reactive protein (CRP). By contrast, eosinophil counts and concentrations of procalcitonin, pro-brain natriuretic peptide (proBNP), D-dimer, creatinine, and lactate differed significantly between the two groups at admission. Lymphocyte counts in BAL fluid were significantly higher in the COP than in the SOP group (*P* = 0.012). Of the 41 (48.2%) patients who underwent PFT, 16 (39.0%) had normal lung function, 12 (29.3%) had a restrictive ventilator defect, nine (22.0%) had an obstructive ventilator defect, and four (9.8%) had mixed defects. PFT results, however, did not differ significantly between these two groups (Table 3).

**Table 3:** Laboratory and clinical findings in patients with cryptogenic and secondary organizing pneumonia

	COP (n = 16)	SOP (n = 69)	Total OP (n = 85)	*P*
PF ratio	297 (270–330) (n = 6)	281 (250–375) (n = 45)	281 (254–367) (n = 51)	0.853
SBP, mmHg	110 (100–120)	120 (110–130)	120 (110–130)	0.100
DBP, mmHg	70 (60–80)	70 (60–80)	70 (60–80)	0.861
PR, /min	85 (76–99)	90 (80–98)	89 (79–99)	0.582
RR, /min	20 (20–20)	20 (20–20)	20 (20–20)	0.401
BT, ℃	37.0 (36.8–37.5)	37.0 (36.7–37.4)	37.0 (36.7–37.4)	0.744
Day at admission				
Leukocyte, 10^3^/ul	6.1 (5.1–9.4)	8.7 (6.6–13.7)	8.7 (6.2–13.6)	0.197
Neutrophil, %	71 (66–79)	80 (72–86)	80 (71–85)	0.088
Eosinophil, %	3 (2–3)	1 (0–2)	1 (0–2)	0.012*
CRP, mg/l	11 (3–49)	108 (24–210)	83 (21–208)	0.067
Procalcitonin, ng/ml	0.05 (0.03–0.05)	0.26 (0.11–1.45)	0.23 (0.08–1.53)	0.018*
proBNP, pg/ml	223 (112–325)	985 (307–2387)	881 (251–2287)	0.035*
D-dimer, ug/ml	0.9 (0.3–1.3)	2.0 (1.3–2.8)	1.9(1.0–2.7)	0.015*
Albumin, g/dl	3.6 (3.3–3.8)	3.3 (3.1–3.7)	3.3 (3.1–3.7)	0.260
Creatinine, mg/dl	0.7 (0.6–0.8)	1.0 (0.7–1.3)	1.0 (0.7–1.3)	0.012*
Lactate, mmol/l	1.0 (1.0–1.1)	1.4 (1.1–1.9)	1.4 (1.0–1.9)	0.047*

Bronchoalveolar lavage	COP (n = 9)	SOP (n = 35)	OP (n = 44)	*P*
Leukocyte, /ul	380 (200–600)	270 (150–850)	300 (150–700)	0.895
Neutrophil, %	7 (4–13)	15 (5–67)	10 (4–56)	0.132
Lymphocyte, %	60 (38–83)	31 (7–56)	36 (9–61)	0.012*
Eosinophil, %	0 (0–1)	1 (1–3)	1 (0–2)	0.007*
Pulmonary function tests**	COP (n = 10)	SOP (n = 31)	OP (n = 41)	0.303
Normal	6 (60.0)	10 (32.3)	16 (39.0)	
Restrictive ventilatory defect	3 (30.0)	9 (29.0)	12 (29.3)	
Obstructive ventilatory defect	1 (10.0)	8 (25.8)	9 (22.0)	
Mixed ventilatory defect	0	4 (12.9)	4 (9.8)	

Data are presented as number (%) or median (interquartile range [IQR])

*COP* cryptogenic organizing pneumonia, *SOP* secondary organizing pneumonia, *OP* organizing pneumonia, *PF* ratio arterial partial pressure of oxygen (PaO_2_)/fraction of inspired oxygen (FiO_2_) ratio, *CRP* C-reactive protein, *proBNP* pro-brain natriuretic peptide, *ESR* erythrocyte sedimentation rate, *FVC* forced vital capacity, *FEV1* forced expiratory volume in the first second, *TLC* total lung capacity, *DLCO* diffusing capacity of the lung for carbon monoxide

*P < 0.05 by Mann–Whitney U-tests for comparisons between the COP and SOP groups

**Classified as normal, restrictive, obstructive, and mixed pattern according to American Thoracic Society criteria [23]

Chest CT showed no between-group differences in the extent of pneumonia, whereas effusion was more common in the SOP group (*P *= 0.036), parenchymal bands or fibrotic pattern and perilobular opacity was more common in the COP group (*P *= 0.005, 0.022, respectively). Chest CT identified 12 patients with focal OP, including seven (43.8%) in the COP group and five (7.2%) in the SOP group (*P* < 0.001) (Table 4).

**Table 4:** Computed tomography findings in patients with cryptogenic and secondary organizing pneumonia, including 12 patients with focal organizing pneumonia

	COP (n = 16)	SOP (n = 69)	Total OP (n = 85)	P
Number of involved lobes				0.185
1	7 (43.8)	19 (27.5)	26 (30.6)	
2	1 (6.3)	16 (23.2)	17 (20.0)	
3	5 (31.3)	9 (13.0)	14 (16.5)	
4	3 (18.8)	10 (14.5)	13 (15.3)	
5	0	15 (21.7)	15(17.6)	
Characters				
Consolidation	5 (31.3)	29 (42.0)	34 (40.0)	0.428
Effusion	2 (12.5)	27 (39.1)	29 (34.1)	0.036*
Both of consolidation and ground glass opacity	4 (25.0)	23 (33.3)	27 (31.8)	0.766
Nodule or mass	6 (37.5)	12 (17.4)	18 (21.2)	0.076
Parenchymal bands or fibrotic pattern	7 (43.8)	9 (13.0)	16 (18.8)	0.005*
Focal solitary nodule	7 (43.8)	5 (7.2)	12 (14.1)	< 0.001*
Cavity	0	10 (14.5)	10 (11.8)	0.196
Mediastinal lymphadenopathy	1 (6.3)	8 (11.6)	9 (10.6)	0.531
Perilobular opacity	4 (25.0)	3 (4.3)	7 (8.2)	0.022*
Ground glass opacity	1 (6.3)	5 (7.2)	6 (7.1)	0.685
Reverse halo sign	1 (6.3)	0	1 (1.2)	0.188

Data are presented as number (%)

*COP* cryptogenic organizing pneumonia, *SOP* secondary organizing pneumonia, *OP* organizing pneumonia

*P < 0.05 by chi-square tests or Fisher’s exact tests for comparisons between the COP and SOP groups

Four patients (5.8%) in the SOP group required ventilator use and five (7.2%) in this group required inotropics, but there were between-group differences. Antibiotic use was significantly higher in the SOP than in the COP group (*P* = 0.013), but there was no between-group difference in steroid use. The 30 day and in-hospital mortality rates, as well as outcome and recurrence rates, did not differ between the two groups (Table 5).

**Table 5:** Treatment and prognosis in patients with cryptogenic and secondary organizing pneumonia

	COP (n = 16)	SOP (n = 69)	Total OP (n = 85)	*P*
Antibiotics	11 (73.3)	65 (94.2)	76 (90.5)	0.013*
Steroid	9 (56.3)	31 (44.9)	40 (47.1)	0.414
Inotropics or vasopressors	0	5 (7.2)	5 (5.9)	0.578
Ventilator	0	4 (5.8)	4 (4.7)	0.427
30 day mortality				0.615
Death	0	3 (4.3)	3 (3.5)	
Loss of follow-up	0	1 (1.4)	1 (1.2)	
In-hospital mortality	0	4 (5.8)	4 (4.7)	0.324
Outcome of OP				0.308
Improvement	14 (89.5)	56 (81.2)	70 (82.4)	
Fail	0	8 (11.6)	8 (9.4)	
Loss of follow-up	2 (12.5)	5 (7.2)	7 (8.2)	
Recur	2 (12.5)	10 (14.5)	12 (14.1)	0.599

Data are presented as number (%)

*COP* cryptogenic organizing pneumonia, *SOP* secondary organizing pneumonia, *OP* organizing pneumonia

*P < 0.05 by chi-square tests for comparisons between the COP and SOP groups

Subgrouping of the 69 patients with SOP cases into those who were (n = 31) and were not (n = 38), treated with steroids showed that pneumonia recurrence was significantly more frequent in the former group (*P* = 0.035). However, there were no differences between these subgroups in 30 day and in-hospital mortality rates and in outcome of OP (Table 6). Median neutrophil counts on complete blood count (CBC) were significantly higher in the recur group (80.2%) than in the non-recur group (66.0%) (*P* = 0.027). Lymphocyte count on CBC was also significantly lower in the recur group (5.6%) than in the non-recur group (7.9%) (*P* = 0.005) (Data not shown).

**Table 6:** Clinical outcomes in patients with SOP who were and were not treated with steroids

	Non-steroid using group (n = 38)	Steroid using group (n = 31)	Total (n = 69)	*P*
OP outcome				
Improve	32 (84.2)	24 (77.4)	56 (81.2)	0.118
Fail	2 (5.3)	6 (19.4)	8 (11.6)	
f/u loss	4 (10.5)	1 (3.2)	5 (7.2)	
In-hospital mortality	1 (2.6)	3 (9.7)	4 (5.8)	0.213
30 day mortality	1 (2.6)	2 (6.5)	3 (4.3)	0.499
Recur	2 (5.3)	8 (25.8)	10 (14.5)	0.035*

Data are presented as number (%)

*SOP* secondary organizing pneumonia, *OP* organizing pneumonia

*P < 0.05 by Fisher’s exact tests for comparisons between the steroid using and non-steroid using SOP subgroups

## Discussion

OP is a pathologic diagnosis term, defined as the presence of means polypoid intraluminal plugs of proliferating fibroblasts and myofibroblasts within alveolar ducts and spaces with varying degrees of bronchiolar involvement. OP can be regarded as a nonspecific pathological pattern of lung responses to injury [6]. In the absence of a specific cause, this condition is diagnosed as COP [9]. The 2002 American Thoracic Society/European Respiratory Society classification recommended using COP rather than the term bronchiolitis obliterans organizing pneumonia (BOOP) because BOOP may be confused with airway disease and, histologically, bronchiolitis obliterans may be concomitant or absent [26]. OP may be a clinical manifestation of a nonspecific repair process in response to a local or distant injury [12]. Based on this definition, SOP was diagnosed in patients with a pathologic OP pattern resulting from identified causes. Distinguishing between COP and SOP is important because the underlying disease, not OP itself, must be managed in patients with SOP. Descriptions of OP are based on an autopsy study of patients with unresolved bacterial pneumonia prior to the advent of antimicrobial therapy [7, 27, 28]. Although OP was thought to be due to infection alone, it was later recognized as a variety of conditions, including interstitial disease, drug toxicity, and connective tissue disorders. Several studies have described the differences between COP and SOP [10–15].

The major difference between these earlier studies [10–15] and the present study is that the SOP group in the latter included a high percentage of patients with infectious pneumonia. Because the clinical symptoms of COP and SOP are similar, it is difficult to diagnose SOP, especially infectious pneumonia, based only on clinical symptoms. In a previous study of 57 patients, 27 were diagnosed with SOP, with eight patients having SOP due to infection, the most frequent cause of SOP in that study [11]. The present study also found that infectious pneumonia was the most frequent cause of SOP. In our center, patients who experience slow clinical improvement after being diagnosed with infectious pneumonia are evaluated by lung biopsy to determine whether other conditions may have accompanied infection. Moreover, many patients with infection in the present study were evaluated by lung biopsy, which may have led to the high rate of infection-related SOP diagnoses. In addition, our study was performed after the development of more recent diagnostic techniques, including culture of respiratory samples, multiplex PCR, and respiratory virus panel tests, enabling more accurate identification of bacteria and viruses, and thus the increased diagnosis of SOP. A study performed from 2001 to 2008 found that only two (9.5%) of 21 patients had SOP caused by infections [12]. However, the difficulty of distinguishing between SOP and COP, and the inability to identify a pathogen, may have resulted in some patients with SOP being diagnosed with COP.

The median age of patients in the present study was 70 years, higher than the average ages reported in previous studies of 50–60 years [10–12, 14]. Moreover, BMI was significantly lower (*P *= 0.030) and the duration of clinical symptoms before admission significantly shorter (*P *= 0.006) in the SOP group. The latter was likely due to the more rapid onset and progression of clinical symptoms in the SOP group, resulting in a shorter duration of symptoms. Among the symptoms, fever was more frequent in the SOP group (*P *= 0.024). A previous study reported that, although most secondary BOOPs were related to chemotherapy and radiotherapy, the clinical symptoms of secondary BOOP were more evident [14]. Our study also found that chronic liver disease was more frequent in the COP group (*P *= 0.020). Advanced liver disease in the pulmonary parenchyma has been reported to lead to lymphocytic or OP [29], which may explain this result. CURB-65, an index of pneumonia severity, was significantly different between COP and SOP, indicating that SOP is more severe than COP (*P *= 0.017).

Serum concentrations of procalcitonin, proBNP, D-dimer, creatinine, and lactate were found to be significantly higher in the SOP than in the COP groups. This increase in SOP was thought to be related to the fact that the main cause of SOP was infectious pneumonia [30] and the severity of SOP was higher than that of COP [31–34]. Lymphocyte counts in BAL fluid were significantly higher in the COP than in the SOP group (*P *= 0.012). Lymphocytosis >25% in BAL fluid is considered a marker of COP [35]. Although a previous study reported that lymphocyte counts in BAL fluid were significantly lower in patients with COP than with SOP, BAL fluid was assessed in only five patients with SOP and 27 patients with COP, with the mean lymphocyte counts in the BAL fluid of the COP group being much lower, 16%, than reported elsewhere [12]. As in previous studies, we found that PFT did not differ between the COP and SOP groups.

CT evaluation showed that pleural effusion was significantly more frequent in the SOP than in the COP group (*P *= 0.036). This finding is in good agreement with the results of previous studies, with effusion being more frequent in patients with SOP than in those with COP [12, 14]. Parenchymal bands or fibrotic pattern and perilobular opacity was more frequent in COP (*P *= 0.005, 0.022, respectively). Parenchymal bands may be secondary to pleuroparenchymal fibrosis [17, 36]. Perilobular opacity is thought to result from residual inflammation in the healing phase [17]. The lesions with surrounding GGO or consolidation are thought to have developed into parenchymal bands, fibrotic pattern or perilobular opacity [36]. Focal OP was more frequent in the COP than in the SOP group (*P* < 0.001). Because focal OP has a different clinical course from other radiologic features of OP [19, 20], focal OP was specifically identified in the present study. This finding differs from the previous study in which the number of focal SOP was greater than the number of focal COP [21]. However, similar to the present study, the previous study showed that symptoms were more frequent in focal SOP than in focal COP, and that the main causes of focal SOP were acute infection and granulomatous inflammation [21].

Antibiotic use was significantly greater in the SOP group (*P *= 0.013), although there was no between-group difference in steroid use. Although COP was found to improve more rapidly with corticosteroids, relapses were more common when treatment was discontinued [8, 13, 24, 37]. Several studies have reported no significant difference in treatment response and clinical course between COP and SOP [10, 12], although one study found a higher mortality rate in patients with SOP, narrowly defined as OP due to a connective tissue disorder, hematologic malignant neoplasms, chemotherapy, or leukemia, than in those with COP [13], suggesting that the underlying disease affects patient prognosis. The present study found no difference in treatment-related outcomes between the COP and SOP groups. Pneumonia recurrence in the SOP group, however, was significantly more frequent in patients who were than were not treated with steroids (*P *= 0.035). This finding was likely due to the low rate of lymphocytosis in the BAL fluid of patients with SOP, which may have reduced responses to steroids and increased the recurrence of pneumonia due to underlying diseases. Among patients with SOP, those who experienced pneumonia recurrence had a significantly higher neutrophil count (*P *= 0.027) and a significantly lower lymphocyte count (*P *= 0.005) than those who did not experience recurrence. High neutrophil counts in patients with infectious SOP cases indicate that infection control is poor, with SOP recurring because of a lack of improvement in the underlying disease.

This study has the following limitations. Because this was a retrospective study of OP diagnosed by lung biopsy, critically ill patients were excluded if lung biopsy was difficult. Because steroids tend to be used only in patients with relatively severe pneumonia, there was likely a selection bias regarding the steroid effect. In this study, steroid dose, duration, and criteria for use were not standardized, making it difficult to interpret treatment-related outcomes. Moreover, recurrence could not be confirmed as a recurrence of OP, as it was not determined histologically by re-biopsy. The frequency of CTD related SOP was low, possibly because patients' symptoms were unintentionally missed, and autoimmune testing for CTD was not confirmed in all patients and only limited testing was performed.

## Conclusions

This study found that the most frequent cause of SOP was infection, with symptoms progressing more rapidly in patients with SOP than with COP. Several parameters in blood and BAL fluid differed significantly in the SOP and COP groups. Pleural effusion was more common in the SOP than in the COP group. PFT results, response to treatment, and treatment results were similar between these two groups. Subanalysis of the SOP group showed that the recurrence rate was higher in those who were than were not treated with steroids.

## Data Availability

All authors had access to data and material and vouch for its complete accuracy. Literature review can be accessed through Pubmed and Google Scholar. Data and materials can be accessed through the medical records at Daegu Catholic University Medical Center. All images are available through PACS imaging system storage at Daegu Catholic University Medical Center.

## Abbreviations

OP: Organizing pneumonia
COP: Cryptogenic organizing pneumonia
SOP: Secondary organizing pneumonia
CTD: Connective tissue disease
BALF: Bronchoalveolar lavage fluid
CT: Computed tomography
BAL: Bronchoalveolar lavage
CRP: Polymerase chain reaction
PFT: Pulmonary function test
TBLB: Transbronchial lung biopsy
AFB: Acid fast bacilli
CoV: Coronaviruses
PIV: Parainfluenza viruses
BMI: Body mass index
CRP: C-reactive protein
Pro-BNP: Pro-B-type natriuretic peptide
CBC: Complete blood count
BOOP: Bronchiolitis obliterans organizing pneumonia
PF ratio: Arterial partial pressure of oxygen (PaO_2_)/fraction of inspired oxygen (FiO_2_) ratio
CRP: C-reactive protein
proBNP: Pro-brain natriuretic peptide
ESR: Erythrocyte sedimentation rate
FVC: Forced vital capacity
FEV1: Forced expiratory volume in the first second
TLC: Total lung capacity
DLCO: Diffusing capacity of the lung for carbon monoxide
